# Association between two common SNPs, rs6564851 and rs6420424, and lutein and zeaxanthin levels in a cohort of US postmenopausal women with a family history of breast cancer

**DOI:** 10.3389/fnut.2024.1372393

**Published:** 2024-10-29

**Authors:** Ann Von Holle, Min Shi, Katie M. O'Brien, Clarice R. Weinberg, Dale P. Sandler, Yong-Moon Mark Park

**Affiliations:** ^1^Biostatistics and Computational Biology Branch, National Institute of Environmental Health Sciences, Durham, NC, United States; ^2^Epidemiology Branch, National Institute of Environmental Health Sciences, Durham, NC, United States; ^3^Department of Epidemiology, Fay W. Boozman College of Public Health, University of Arkansas for Medical Sciences, Little Rock, AR, United States; ^4^Winthrop P. Rockefeller Cancer Institute, University of Arkansas for Medical Sciences, Little Rock, AR, United States

**Keywords:** carotenoids, lutein, zeaxanthins, genome-wide association study, genotype, BCO1

## Abstract

A better understanding of the factors contributing to systemic concentrations of carotenoids is necessary given the weak correlations between circulating levels and dietary intake of carotenoids. Although genetic variation may play a key role in the interindividual variability in carotenoid concentrations, few genome-wide association studies (GWAS) have focused on carotenoids. We used a random sample (*n* = 519) of postmenopausal participants in the Sister Study with data on genotypes and plasma carotenoid levels to conduct GWAS for each of five carotenoids (mcg/mL): alpha-carotene, beta- carotene, cryptoxanthin, lycopene, and lutein/zeaxanthin. We used linear regression models and an additive genetic model to evaluate associations between 371,532 variants and inverse normal transformed carotenoid concentrations. We found evidence for one genome-wide statistically significant association with the combined carotenoids of lutein and zeaxanthin for rs6564851-C (beta = −0.377, se = 0.059, *p* = 4.6×10^−10^) and rs6420424-A (beta = −0.334, se = 0.059, *p* = 2.2×10^−8^), upstream of beta-carotene oxygenase 1 (BCO1) gene on chromosome 16. No other variant was associated with any of the remaining four carotenoids. Our results for the common rs6564851 and rs6420424 variants correspond to previous findings. Although biologic mechanisms explain the association between beta-carotene and the variants, the inverse association with lutein/zeaxanthin will require further investigation.

## 1 Introduction

Carotenoids are a group of lipophilic pigmented compounds produced by plants and microorganisms but not by humans (1). Dietary intake of fruits and vegetables is the primary source of carotenoids, including *α*-carotene, *β*-carotene, β-cryptoxanthin, lutein/zeaxanthin, and lycopene (2). Consumption of carotenoids and circulating carotenoids are associated with decreased risk of several outcomes including obesity (3, 4), cardiometabolic disease (5, 6), some cancers (7–10), eye diseases (11, 12), and mortality (13, 14).

Dietary intake of carotenoids is poorly correlated with plasma levels because of measurement errors in carotenoid consumption from self-administered dietary questionnaires and variations in bioavailability of carotenoids from different foods. Biomarker measurements of circulating carotenoids may be more reflective of underlying carotenoid exposure, although they too are subject to measurement error (15). Furthermore, concentrations of circulating carotenoids by individual differences in absorption and metabolism of carotenoids, may be influenced by the degree of food processing in the source of the carotenoids and genetic factors (16).

Genetic variation is suggested to play a key role in the interindividual variability in carotenoid concentrations (17, 18). In addition, the effect of genetic variants on plasma concentrations of carotenoids may affect the ability of carotenoids to prevent chronic diseases (19). Several single nucleotide polymorphisms (SNPs) are known to be associated with circulating carotenoid status, but the evidence has been limited to mostly candidate gene association studies (17). A few genome-wide association studies (GWAS) showed that the *β*-carotene 15,15′-monooxygenase 1 (BCMO1) gene affects circulating carotenoid levels (20) and observed significant associations with *α*-carotene concentrations for three novel loci (21).

Thus, we aimed to identify genome-wide associations with plasma carotenoids using a subcohort sample (*n* = 513) from a previous case-cohort study of carotenoids and postmenopausal breast cancer from the Sister Study (22).

## 2 Methods

### 2.1 Sample

Data were obtained from the Sister Study, a prospective cohort of 50,884 women who had a sister with breast cancer, but had not been diagnosed with breast cancer themselves prior to baseline (23). Participants aged 35–74 years in the United States, including Puerto Rico, were enrolled between 2003 and 2009. At enrollment, anthropometric measurements and biological samples, including blood samples, were taken by trained examiners in a home exam. The Sister Study is overseen by the National Institutes of Health Institutional Review Board. All participants provided written informed consent.

We previously assessed carotenoid levels in plasma taken from a random sample of 524 Sister Study participants who were postmenopausal at enrollment (22). The random sample included 43 women who developed breast cancer after enrollment. Carotenoids measured included alpha-carotene, beta-carotene, cryptoxanthin, lycopene, and lutein/zeaxanthin, all measured in mcg/mL. We combined lutein and zeaxanthin because zeaxanthin is a structural isomer of lutein, and they have similar health effects (24). We also examined a second internal validation sample of estrogen receptor (ER)-negative and ER-positive breast cancer cases (n = 400) that were also sampled for the original carotenoid and oxidative stress analysis and had genotype data. The data was from release 9.1, with follow-up through 9/30/2019. Measurement of these fasting plasma carotenoids has been described (22). In brief, these five carotenoids were analyzed across 64 batches with control samples using high-performance liquid chromatography (HPLC) and calibration relying on standards within physiological ranges and corrected for HPLC purity. Carotenoid concentrations were adjusted for batch effects by subtracting batch-specific random effects from the measured level.

Genotyping of blood samples collected at enrollment was conducted using the Infinium OncoArray genotyping panel (Illumina Inc.) (25). The OncoArray panel has more than 530,000 single nucleotide polymorphisms (SNPs), including ancestry informative markers. Around half of the SNPs had been included in that panel to create a GWAS backbone, and the other half had been included as markers for specific sites known or hypothesized to be cancer-related.

Given the random sample with a combination of a fully characterized set of carotenoids and genotype data from a large commonly used panel, we conducted a GWAS to assess associations between carotenoids and SNPs. This exploratory study with a relatively smaller sample size may inform and confirm existing associations between carotenoid levels with SNPs having a larger minor allele frequency (MAF)—SNPs with a smaller MAF are not as likely to demonstrate a signal given the sample size.

### 2.2 Statistical methods

Descriptive statistics for each sample included the median and interquartile ranges (IQR) for continuous variables and percentages with corresponding counts for categorical variables.

We used PLINK (v1.9) (26, 27) to conduct quality control for the discovery sample with 523 participants with at least one carotenoid measure and genotype data for at least 494,444 SNPs. Quality control included the following steps: (a) deleting SNPs with more than 2% missing (*n* = 993), (b) removing individuals missing more than 2% of genotype data (*n* = 4), (c) removing non-autosomal SNPs (*n* = 26,232), (d) removing SNPs with minor allele frequency < 0.02 (*n* = 95,687). We did not remove any people from the sample based on relatedness and there was one family in the sample with two sisters participating in the study. After the quality control process, 519 individuals and 371,532 autosomal SNPs remained in the discovery sample. Given that the participants in our sample had a first-degree family history of breast cancer, we calculated, but did not exclude, SNPs based on Hardy Weinberg equilibrium (HWE) *p*-values.

In the internal validation sample based on 402 remaining cases with at least one non-missing carotenoid value and genotype data for 494,444 SNPs, the quality control process was as follows: (a) deleting SNPs with missingness >0.02 (*n* = 809), (b) removing individuals missing more than 2% of genotype data (*n* = 4), (c) removing non-autosomal SNPs (*n* = 26,251), (d) removing SNPs with minor allele frequency < 0.02 (*n* = 95,008). Of the remaining 372,376 SNPs in the validation sample, there were 366,482 SNPs also present in the discovery sample, which we used for analyses. After the quality control process, 398 individuals and 366,482 SNPs remained in the validation sample.

To account for population stratification, we calculated principal component values using PLINK and based on 2,290 ancestry informative markers (25). The top five components were adjusted for in each multivariable model.

Linear regression models in PLINK were used to test genetic associations with each of the continuous carotenoid levels. After evaluating QQ-plots and a range of transformations, we used the inverse normal transform (28) for all the carotenoids, given the extreme values present in the distribution of these outcomes. This transformation involves finding the sample quantile and back-transforming to a standard normal score. In the linear regression model, we assumed an additive effect of allele count at each SNP. In addition to the first five ancestry principal components, we included age at blood draw and examiner-measured body mass index (BMI) at enrollment as covariates in the linear regression models. To account for false discoveries in the numerous association tests, we used a genome-wide Bonferroni testing threshold of *p* < 5 × 10^−8^ for an alpha level of 0.05. Data visualization of the association tests included Manhattan plots and quantile-quantile (QQ) plots. With the QQ-plots, we also estimated the lambda statistic, the genomic inflation factor, to assess bias. Following association tests, we used LocusZoom (29) to provide regional information for SNPs below our *p*-value threshold.

## 3 Results

The primary and validation samples had similar medians and IQR for each of the carotenoids (Table 1). The median BMI of participants in the primary sample was 27 kg/m^2^, and the median age at enrollment was around 60 years. The carotenoid levels in our analytic sample were all positively correlated with one another (Supplementary Figure 1), with the strongest correlation being 0.69 for beta-carotene and alpha-carotene and the weakest correlation being 0.25 for alpha-carotene and lycopene.

**Table 1:** Descriptive statistics by sample.

Characteristic	Primary, *N* = 519	Replication, *N* = 398
Total carotenoids, Median (IQR)	1.30 (0.96–1.77)	1.31 (0.99–1.80)
Alpha carotene, Median (IQR)	0.08 (0.05–0.12)	0.08 (0.05–0.13)
Beta Carotene, Median (IQR)	0.36 (0.23–0.57)	0.37 (0.22–0.58)
Lycopene, Median (IQR)	0.42 (0.32–0.58)	0.42 (0.32–0.56)
Cryptoxanthine, Median (IQR)	0.11 (0.07–0.17)	0.11 (0.07–0.18)
Lutein and Zeaxanthin, Median (IQR)	0.27 (0.19–0.38)	0.27 (0.20–0.36)
Lutein, Median (IQR)	0.20 (0.14–0.29)	0.21 (0.14–0.28)
Zeaxanthin, Median (IQR)	0.07 (0.05–0.09)	0.07 (0.05–0.09)
BMI, kg/m^2^, Median (IQR)	27 (23–31)	28 (24–32)
Age (years), Median (IQR)	60 (56–65)	61 (56–66)
Race/ethnicity, n (%)		
Non-Hispanic white	457 (88)	343 (86)
Non-Hispanic Black	33 (6.4)	26 (6.5)
Hispanic	12 (2.3)	16 (4.0)
Other	17 (3.3)	13 (3.3)

**Figure 1:**
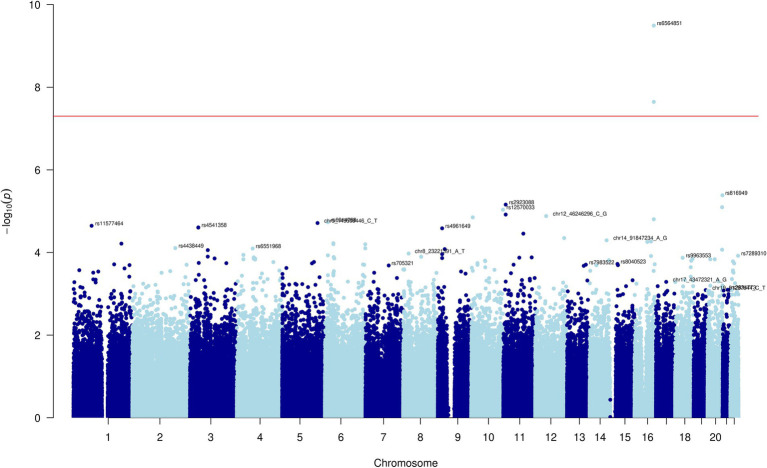
Manhattan plot for lutein and zeaxanthin carotenoid.

After conducting the genome-wide age and BMI adjusted association tests for the five carotenoids, we observed two SNPs with genome-wide statistically significant associations with the combined category of lutein and zeaxanthin: rs6564851 and rs6420424 on chromosome 16 (Figure 1). For each additional copy of the C allele (allele frequency = 0.52) for rs6564851, there was a 0.381 (SE = 0.059, *p*-value = 1.9×10^−10^) standard deviation decrease in the inverse normal transformed combined lutein and zeaxanthin concentration (Table 2). The median batch-adjusted values of the lutein and zeaxanthin concentrations by genotype confirm this association (Supplementary Figure 2; Supplementary Table 1).

**Table 2:** Association tests for rs6564851 and rs6420424, chromosome 16.

SNP/Carotenoid	Position	Index/reference allele	Beta	SE	*p*-value
rs6420424					
Lutein and Zeaxanthin	81242102	A/G	−0.334	0.059	2.24281e-08
Lutein	81242102	A/G	−0.327	0.059	4.55187e-08
Zeaxanthin	81242102	A/G	−0.289	0.061	2.60106e-06
Beta carotene	81242102	A/G	0.131	0.059	0.0265754
rs6564851					
Lutein and Zeaxanthin	81264597	C/A	−0.381	0.059	1.86233e-10
Lutein	81264597	C/A	−0.372	0.059	4.73324e-10
Zeaxanthin	81264597	C/A	−0.339	0.061	3.60168e-08
Beta carotene	81264597	C/A	0.148	0.059	0.0123591

For each additional copy of the A allele (allele frequency = 0.50) of the rs6420424 SNP there was a 0.334 (SE = 0.059, *p*-value = 2.2×10^−8^) standard deviation decrease in the inverse normal transformed combined lutein and zeaxanthin concentration (Table 2). We confirmed this association in the estimates of median lutein and zeaxanthin concentrations by genotype (Supplementary Table 2). We found evidence that rs6420424 is in LD (r^2^ = 0.66, *D* = 0.84) with the top SNP, rs6564851.

A *post-hoc* analysis of the separate lutein and zeaxanthin outcomes for rs6564851 suggested both carotenoids contribute to this association. The additive association for the C allele was −0.372 (SE = 0.059, *p*-value = 4.7×10^−10^) for lutein and − 0.339 (SE = 0.061, *p*-value = 3.6×10^−8^) for zeaxanthin (Table 2). Given that the medians were similar for the AA and AC genotypes, we also conducted a post-hoc analysis using a recessive model for the rs6564851 SNP (Supplementary Table 3).

**Figure 2:**
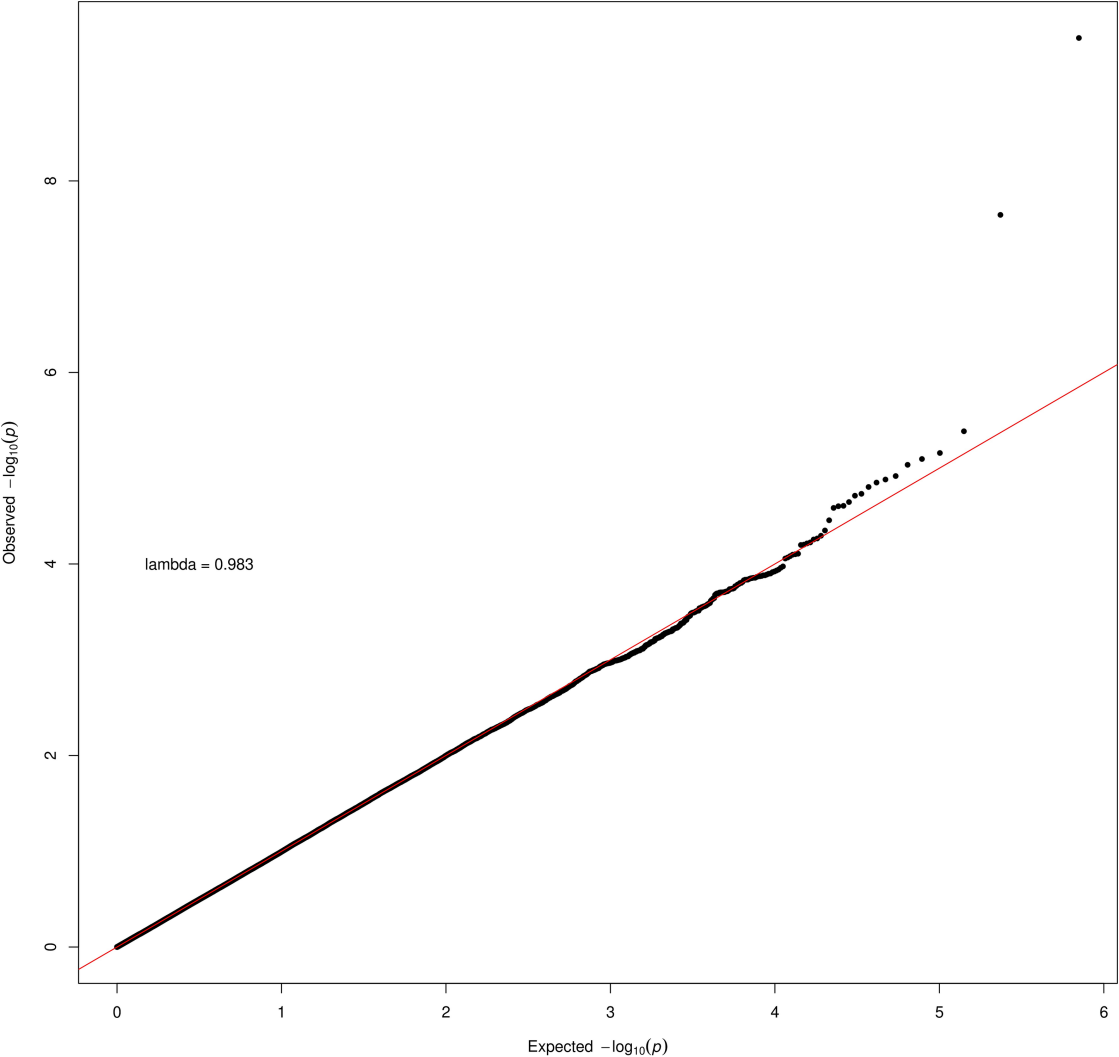
QQ plot for lutein and zeaxanthin carotenoid.

Inspection of the QQ plot (Figure 2) for the transformed data shows little evidence of inflation in the association statistic (genomic inflation factor, lambda = 0.983). Inspection of the rs6564851 and rs6420424 variants in a regional association plot shows these intergenic variants are upstream of the beta-carotene oxygenase 1 (BCO1) gene (Supplementary Figure 3).

We did not find evidence of any genome-wide statistically significant associations for the other four carotenoids (Supplementary Figures 4–15). The association in the validation sample was also negative for the C allele of rs6564851 with a decrease in lutein and zeaxanthin (beta = −0.184, SE = 0.065, *p*-value = 0.005; Supplementary Table 4). Similarly, for the A allele of rs6420424 there was a decrease in lutein and zeaxanthin (beta = −0.139, SE = 0.068, *p*-value = 0.041).

## 4 Discussion

We conducted GWAS for five carotenoids in a sample of over 500 U.S. women to discover variants associated with alpha-carotene, beta-carotene, cryptoxanthin, lycopene, and combined lutein and zeaxanthin. There was a genome-wide statistically significant association between two variants, rs6564851 (negative for the C allele) and rs6420424 (negative for A allele), and the combined lutein and zeaxanthin levels. We qualitatively replicated this association in a distinct sample of cases from the same data source. In separating out the two carotenoids, we found similar associations showing that both lutein and zeaxanthin contributed to the significant negative associations with the combined levels.

The rs6564851 and rs6420424 variants have been discussed in the literature in relation to beta-carotene serum levels (20, 30–32) in non-US samples, but not specifically as relative to lutein and zeaxanthin levels. In a GWAS (20) that assessed the same carotenoids, the investigators found inverse, additive associations (95% CI) between the G allele of rs6564851 (for this comparison, C and G alleles considered the same variant), the SNP most strongly associated with beta carotene, and lutein [−0.032 (−0.040, −0.024)] and zeaxanthin [−0.008 (−0.012, 0.004)], with the lutein association attaining genome-wide statistical significance. In this same study, a haplotype-based analysis including rs6564851 and rs6420424 demonstrated similar effect sizes similar to rs6464851 alone. Also, in this study, the G allele from rs6564851 was positively associated with beta-carotene [0.149 (95% CI: 0.120, 0.177)]. Our results replicate this association found between this SNP and lutein. Although not significant at a genome-wide level, we found an additive association (95% CI) with the C allele for the inverse normal transformed beta-carotene level (0.148, SE = 0.059, *p* = 0.01). As previously discussed (20), the direction of association is not the same for each of these carotenoids, and the mechanism underlying this difference remains unexplained.

The rs6564851 and rs6420424 variants are upstream from the BCO1 gene, which codes a key enzyme in beta-carotene metabolism to vitamin A. As noted in a prior study (20), both variants are also within a 23 kb region including the Polycystic kidney disease 1-like 2 (PKD1L2) gene, but the BCO1 gene represents a better candidate for further exploration. Functional investigation of these variants reveals they are in a promoter region (33) of BCO1 associated with the regulation of vitamin A production. The rs6564851 and rs6420424 variants were associated with catalytic activity of BCO1 (30), also associated with beta-carotene concentrations. A prior finding that female carriers of the T allele of rs6564851 and G allele of rs6420424 demonstrated a reduction in beta-carotene conversion (32) that is consistent with our results despite its different population of young female volunteers with a mean age of 20 years and a smaller sample size (*n* = 28). Our findings show an association between this variant and beta-carotene that corresponds to the direction of effect outlined in the prior study, but it was not statistically significant at a genome-wide level (Table 2).

There is no biologically plausible function related to lutein and zeaxanthin based on prior research with this variant and the proximal BCO1 gene. Potential explanations for the association between this region and lutein and zeaxanthin levels include the role of beta-carotene levels that are associated with this region and influencing the lutein and zeaxanthin levels on a separate unstudied physiological path (20). This interpretation suggests beta carotene influences lutein and zeaxanthin through its relationship with the rs6564851 and rs6420424 variants instead of a direct association between the variant and lutein and zeaxanthin. However, if higher beta-carotene levels suppress lutein and zeaxanthin levels, the positive correlation between these observed carotenoids in our analytic sample (Supplementary Figure 1) does not correspond to a hypothesis of higher beta-carotene levels suppressing lutein and zeaxanthin levels. Pleiotropy, the separate associations between a SNP and multiple phenotypes, could also explain these different associations with beta-carotene and lutein and zeaxanthin.

Among the advantages of this work, the number of carotenoids allowed us to assess a broad array of carotenoids in this GWAS. With these outcomes, we confirmed an association not extensively assessed in the literature between lutein and zeaxanthin.

Several limitations exist for this study. One limitation was the selection of only postmenopausal women due to the scientific rationale of the parent study from which our sample was drawn, representing a random selection from a case-cohort study to investigate associations between carotenoids and inflammation (22). Another limitation of these analyses was the small sample size, which restricts our power to detect smaller effect sizes as statistically significant. Also, the validation sample that included cases from the same study was not an independent sample and is a limitation of this work. However, the results from the convenient validation sample matched our findings from the discovery sample in terms of the direction of the association. Replication of these results and further study of functional effects can further our understanding of these differing associations between these two carotenoids and the rs6564851 and rs6420424 variants.

Findings from this study may have implications for carotenoid research, particularly in areas such as Mendelian randomization (MR) and nutritional supplementation. We identified one SNP associated with lutein and zeaxanthin, rs6564851, which has been included in MR studies investigating associations between beta carotene and outcomes such as cardiovascular disease (34) and inflammatory bowel disease (35). Future MR studies may also include rs6564851 as an instrumental variable for lutein and zeaxanthin levels. Identifying these carotenoids, in addition to beta-carotene, and their association with this SNP could provide information on causal pathways for these health outcomes. Furthermore, another study found higher plasma beta-carotene levels associated with the G allele of the rs654851 SNP in Ghana (30) and suggested that it may be relevant to food fortification efforts in populations at risk for malnutrition. Given the association with lutein and zeaxanthin in our results, studies investigating supplementation with lutein and zeaxanthin may also need to consider this variant and its potential for reduced efficacy in certain groups of people.

In summary, we have found an association between two common SNPs, rs6564851 and rs6420424, and combined serum lutein and zeaxanthin levels, which is consistent with a prior study (20). Although we did not replicate a previous discovery of an inverse association with this variant and beta-carotene levels at a genome-wide significance level, we found a similar inverse direction of association relative to the association with lutein and zeaxanthin. Biologic mechanisms explaining the association between the rs6564851 and rs6420424 variants and lutein and zeaxanthin levels remain unclear and further investigation is needed to understand this relationship. These common variants may be important when assessing circulating serum levels of lutein and zeaxanthin.

## Data Availability

The Sister study genotype data was deposited as part of NCI's Genetic Associations and Mechanisms in Oncology (GAME-ON) initiative. You can find this information under the following accession id: dbGaP Study Accession: phs001265.v1.p1.
