# Supplementary material for: Association between two common SNPs, rs6564851 and rs6420424, and lutein and zeaxanthin levels in a cohort of US postmenopausal women with a family history of breast cancer
Source: Front Nutr. 2024 Oct 29;11:1372393. doi: 10.3389/fnut.2024.1372393 (PMC11554543; doi:10.3389/fnut.2024.1372393)
Supplement: Supplementary file 1 [file Data_Sheet_1.PDF]

Supplementary tables and figures

Supplementary Table 1: Carotenoid characteristics by rs6564851 genotype

Characteristic	N	rs6564851 genotype AA N = 113	AC N = 273	CC N = 133
Lutein and Zeaxanthin	519	0.29 (0.23, 0.42)	0.29 (0.20, 0.40)	0.22 (0.16, 0.29)
Lutein	519	0.22 (0.18, 0.33)	0.21 (0.15, 0.31)	0.16 (0.11, 0.23)
Zeanxanthin	519	0.07 (0.05, 0.10)	0.07 (0.05, 0.09)	0.06 (0.04, 0.07)

[1] Median (Q1, Q3)

Supplementary Table 2: Carotenoid characteristics by rs6420424 genotype

**Characteristic**	**N**	rs6420424 genotype<br>**GG**<br>N = 121	 **AG**<br>N = 275	 **AA**<br>N = 123
Lutein and Zeaxanthin	519	0.30 (0.23, 0.43)	0.27 (0.19, 0.37)	0.22 (0.16, 0.32)
Lutein	519	0.23 (0.17, 0.35)	0.20 (0.14, 0.29)	0.16 (0.11, 0.24)
Zeanxanthin	519	0.08 (0.06, 0.10)	0.07 (0.05, 0.09)	0.06 (0.04, 0.08)

[1] Median (Q1, Q3)

Supplementary Table 3: Recessive association tests for rs6564851, chromosome 16, position 81264597, index allele C.

Carotenoid	Beta	SE	p-value
Lutein and Zeaxanthin	-0.558	0.092	3.02545e-09
Lutein	-0.539	0.093	1.05858e-08
Zeaxanthin	-0.506	0.095	1.66241e-07
Beta carotene	0.252	0.093	0.0066614

Supplementary Table 4: Association tests for rs6564851 and lutein and zeaxanthin in case-only sample

Carotenoid	Chromosome	Position	Index allele	Beta	SE	p-value
**rs6420424**						
Lutein and Zeaxanthin	16	81242102	A	-0.139	0.068	0.041
Lutein	16	81242102	A	-0.139	0.069	0.043
Zeaxanthin	16	81242102	A	-0.085	0.069	0.219
Beta carotene	16	81242102	A	0.176	0.069	0.011
**rs6564851**						
Lutein and Zeaxanthin	16	81264597	C	-0.184	0.065	0.005
Lutein	16	81264597	C	-0.176	0.066	0.008
Zeaxanthin	16	81264597	C	-0.154	0.066	0.019
Beta carotene	16	81264597	C	0.164	0.066	0.013

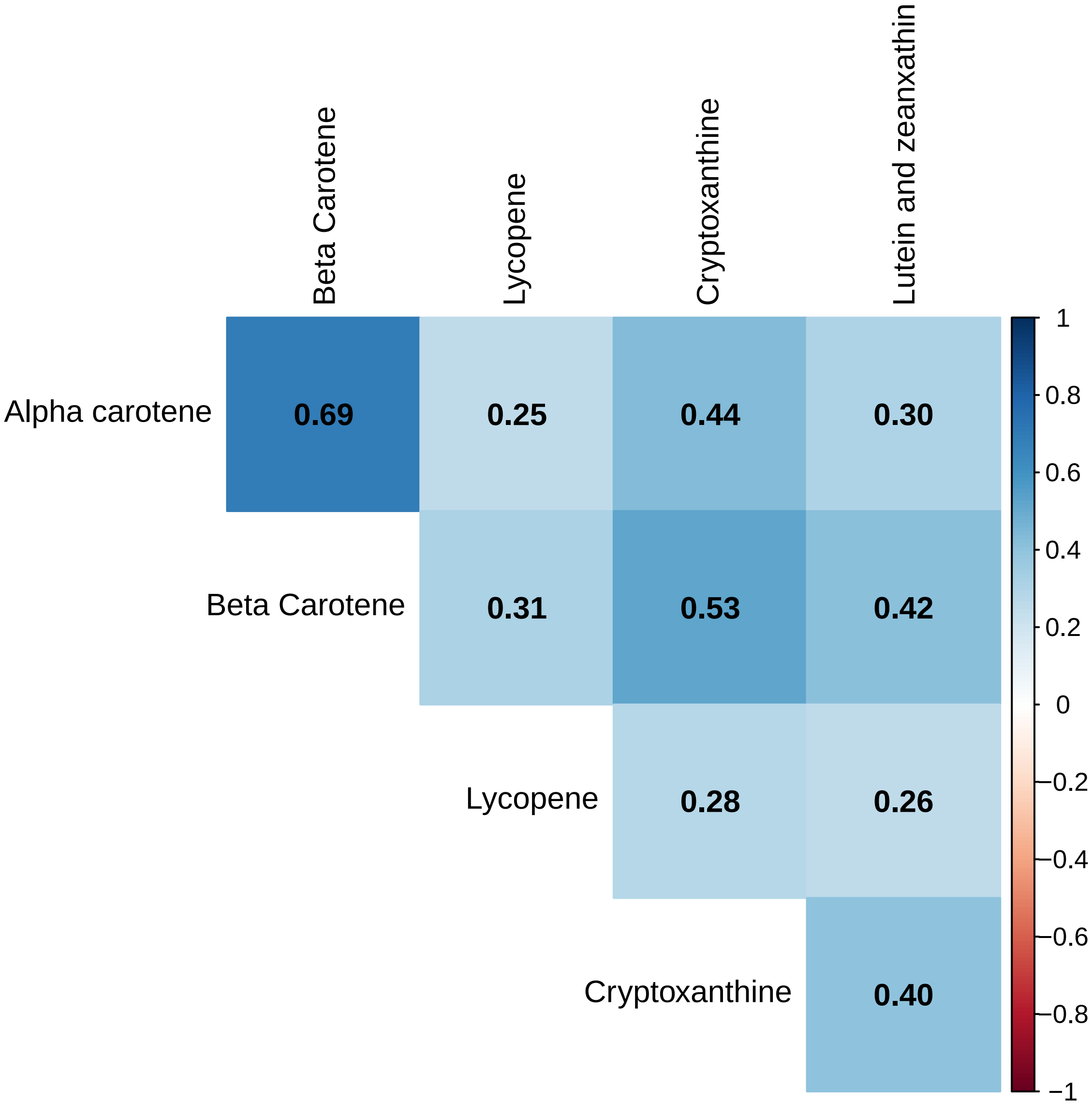

Supplementary Figure 1: Correlation heat map of five primary carotenoids

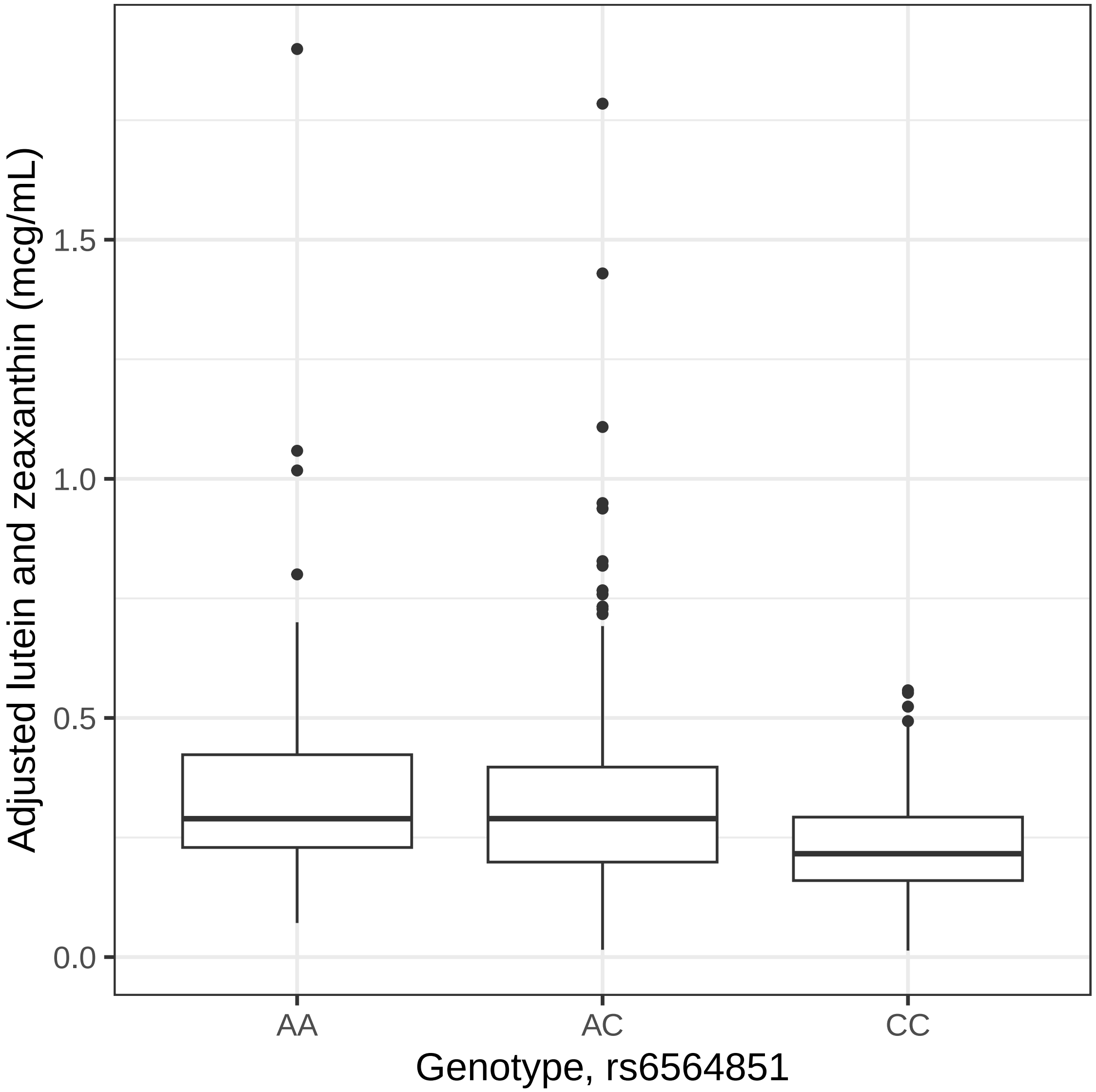

Supplementary Figure 2: Boxplot of Lutein and Zeaxanthin by rs6564851 genotype

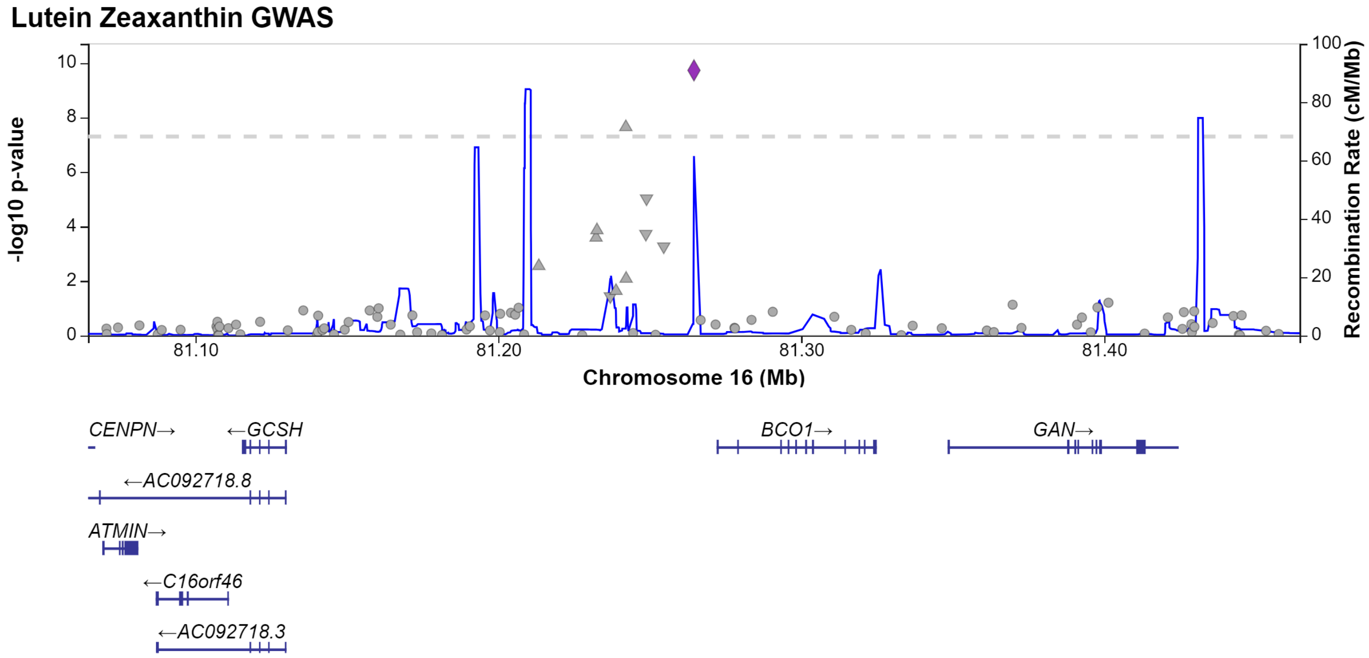

Supplementary Figure 3: Regional association plot for variants rs6564851 and rs6420424

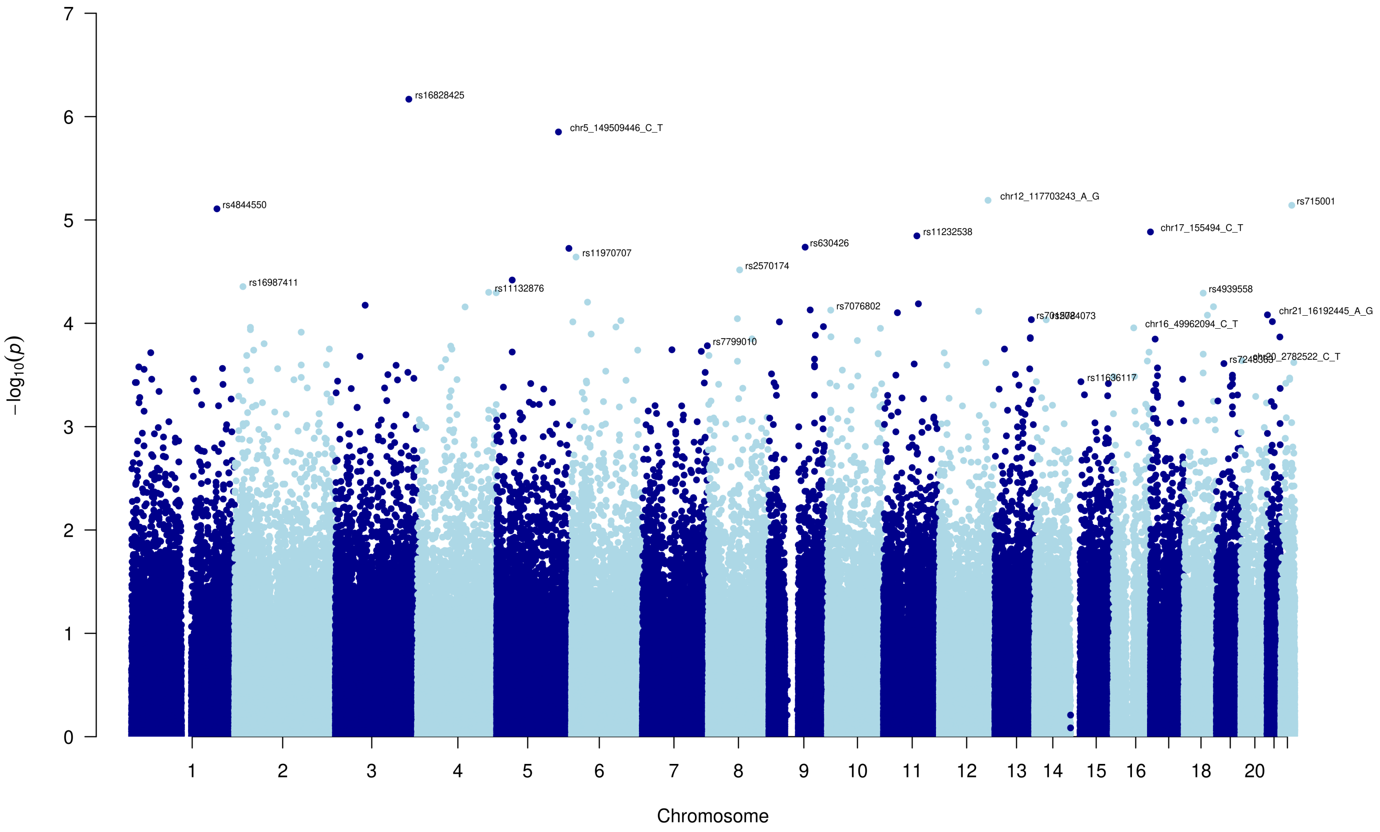

Supplementary Figure 4: Manhattan plot for alpha carotene, primary sample

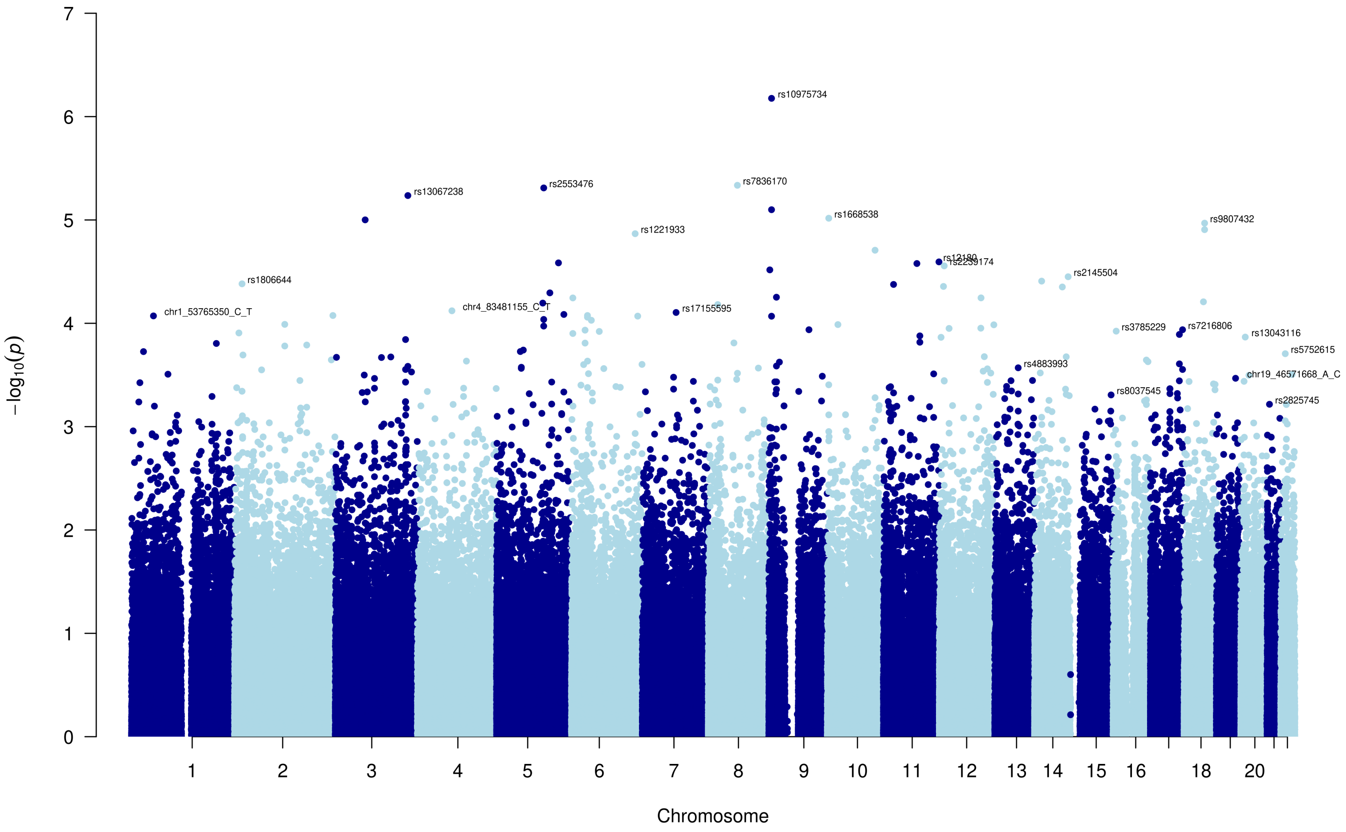

Supplementary Figure 5: Manhattan plot for beta-carotene, primary sample

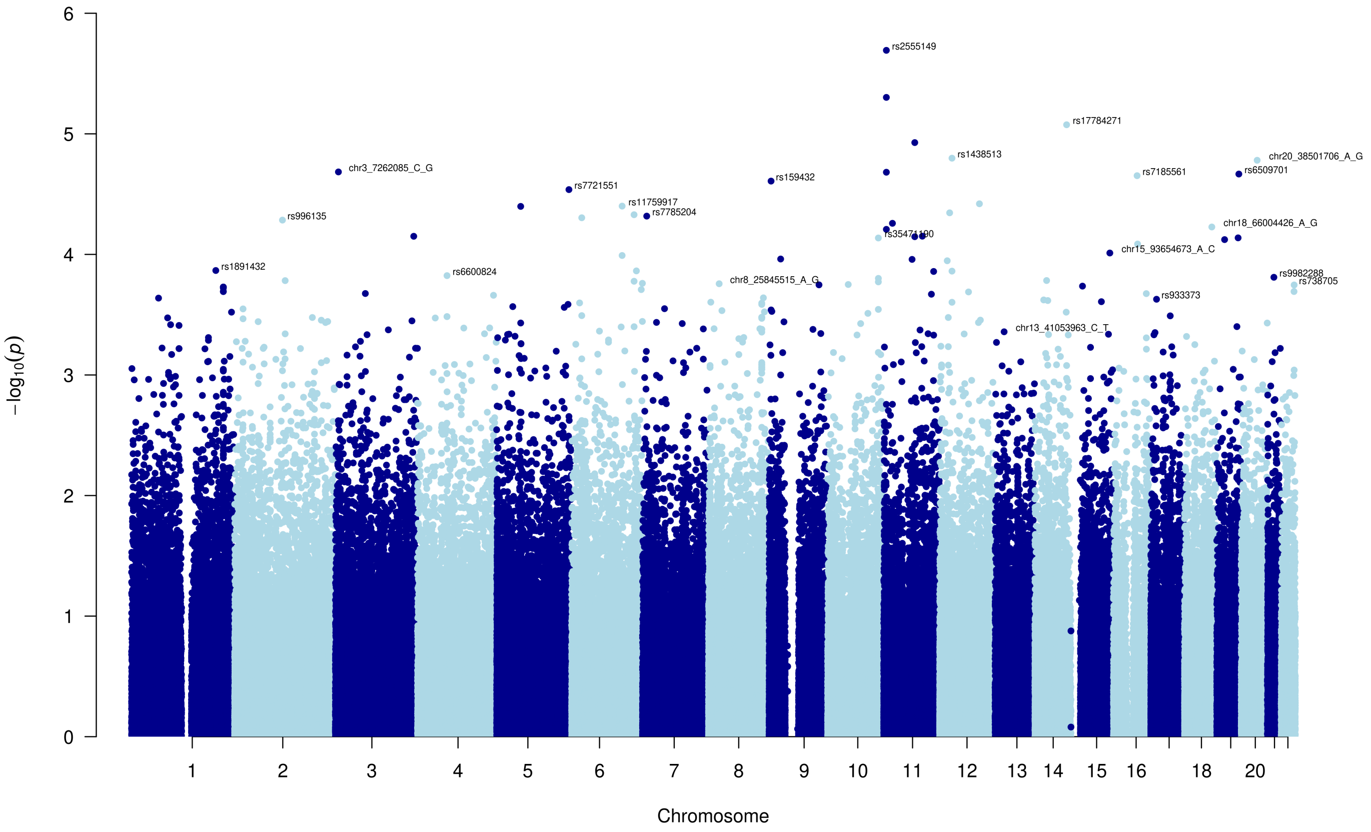

Supplementary Figure 6: Manhattan plot for lycopene, primary sample

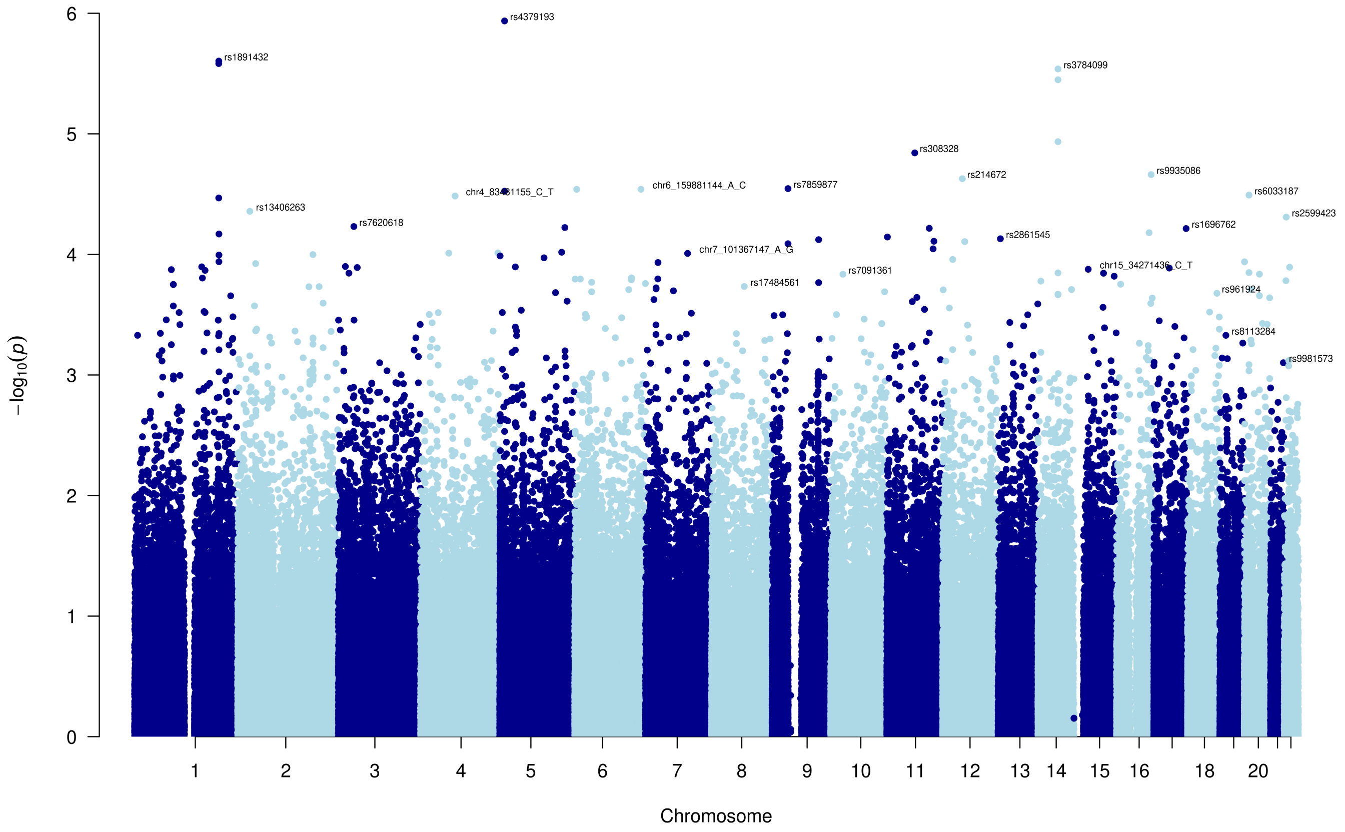

Supplementary Figure 7: Manhattan plot for cryptoxanthin, primary sample

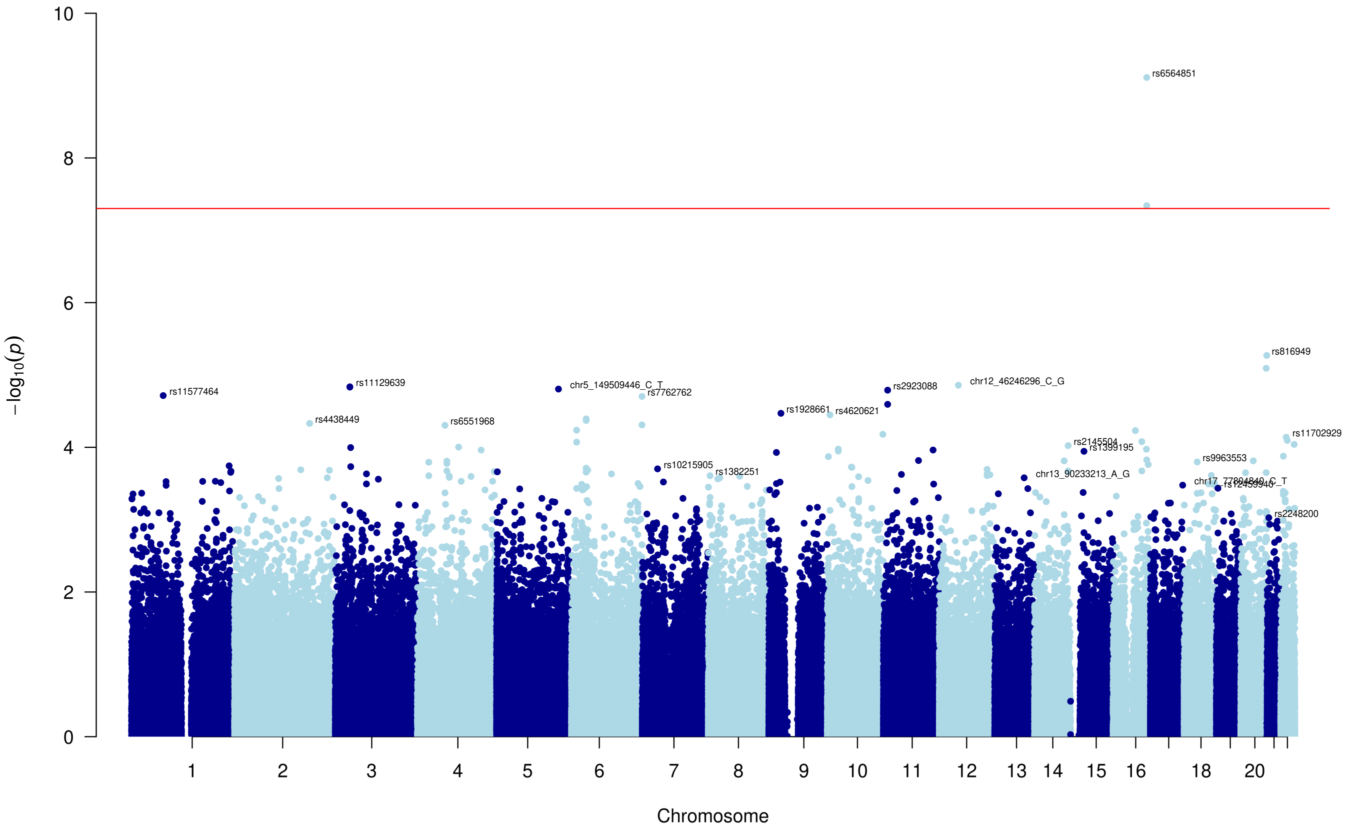

Supplementary Figure 8: Manhattan plot for Lutein, primary sample

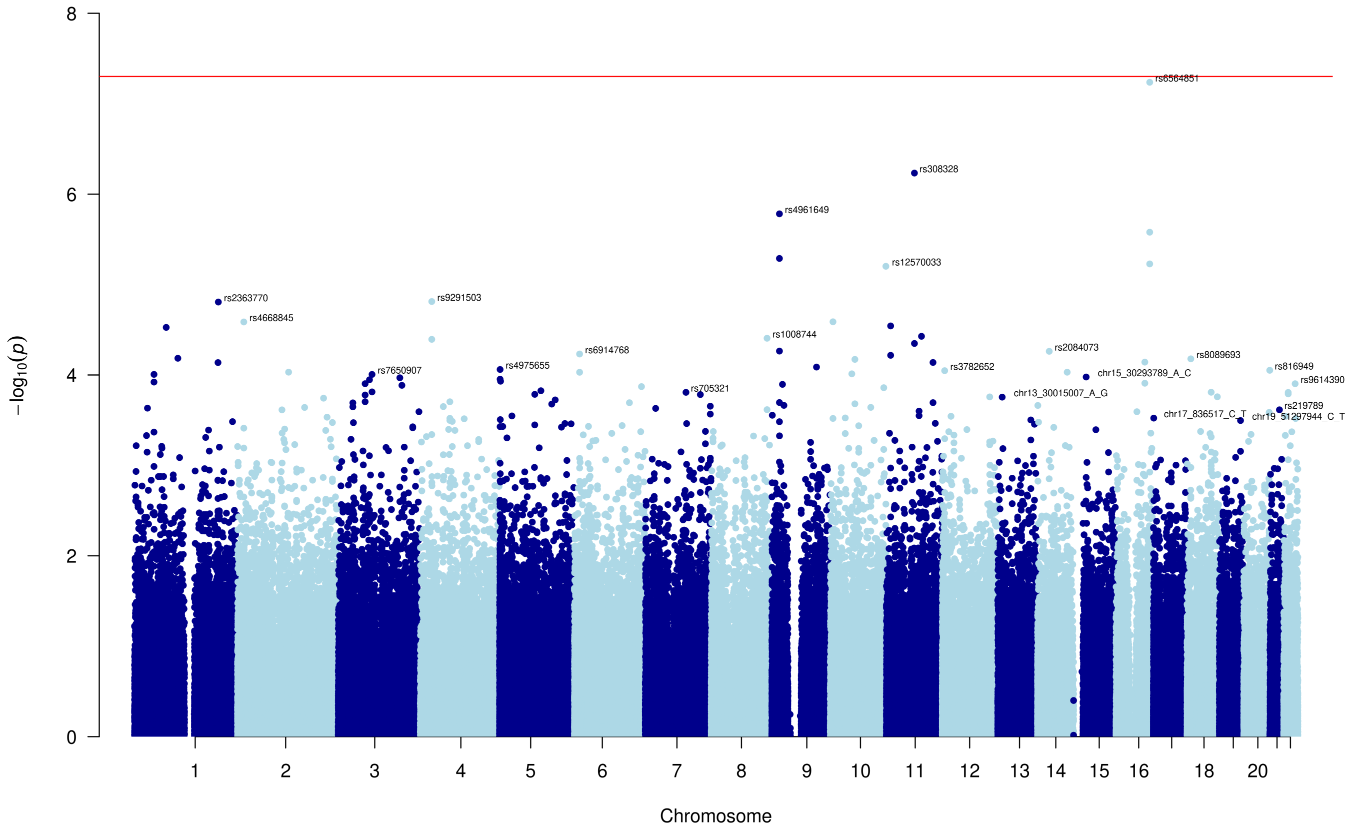

Supplementary Figure 9: Manhattan plot for Zeaxanthin, primary sample

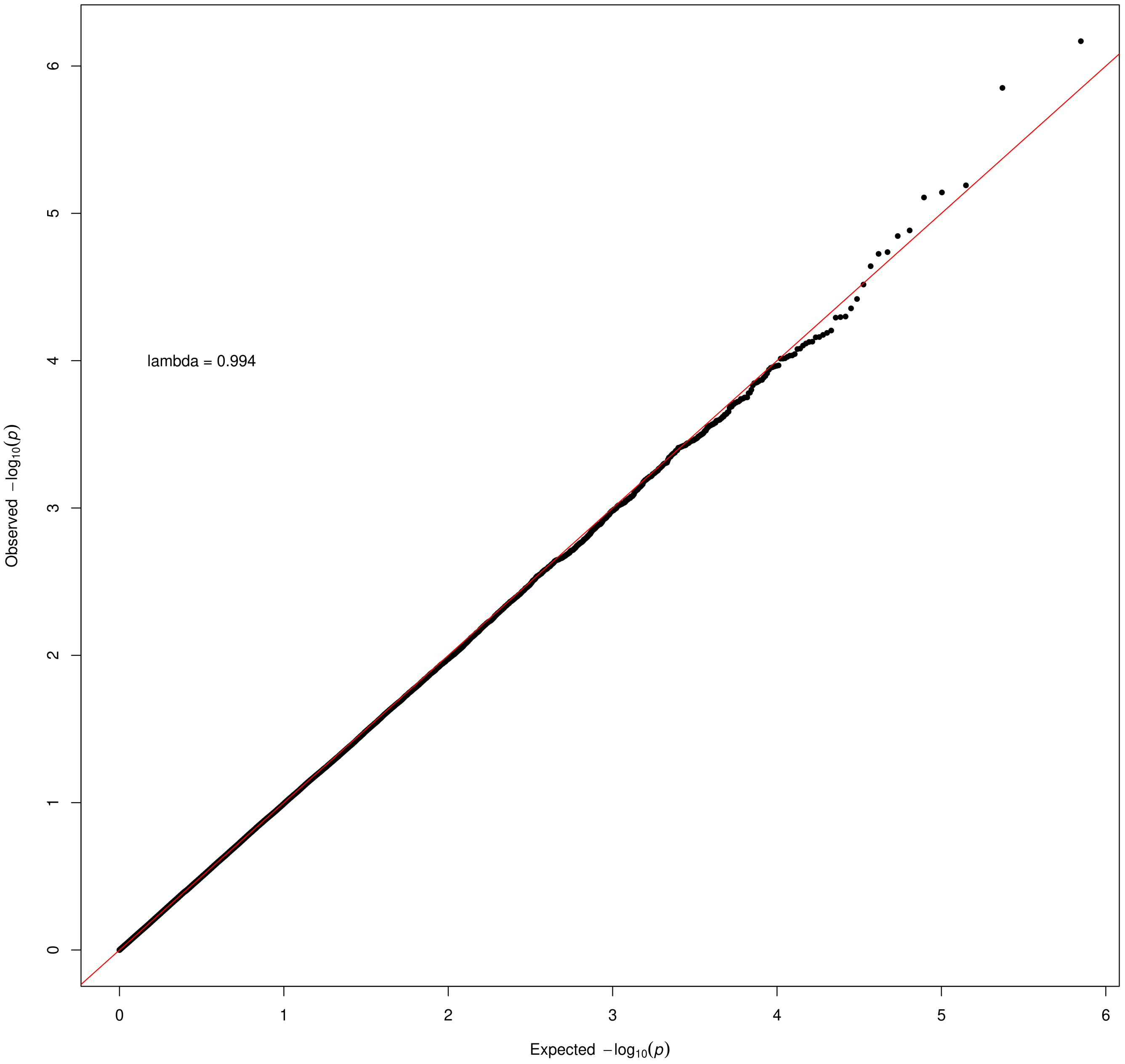

Supplementary Figure 10: QQ plot for alpha carotene, primary sample

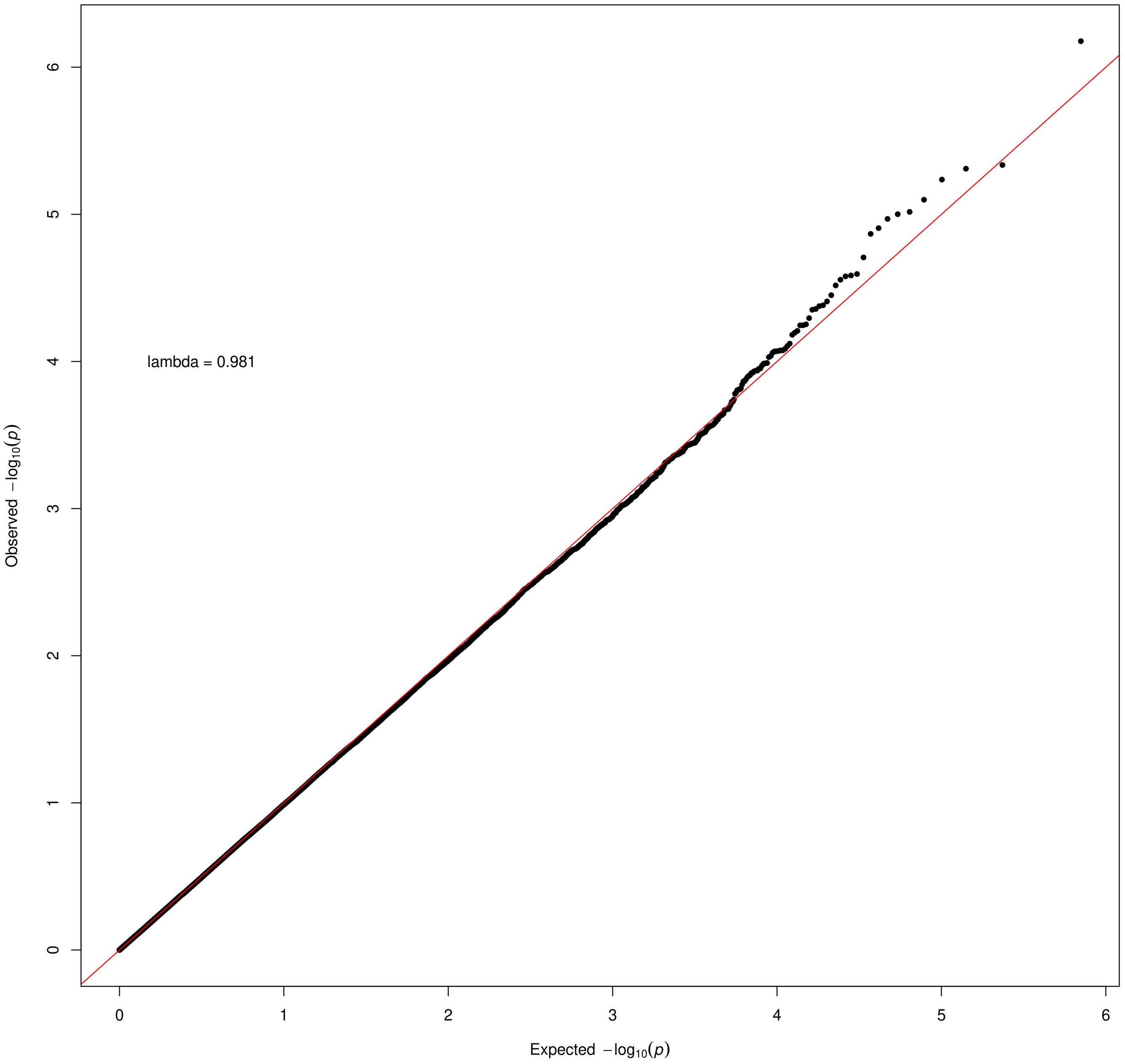

Supplementary Figure 11: QQ plot for beta-carotene, primary sample

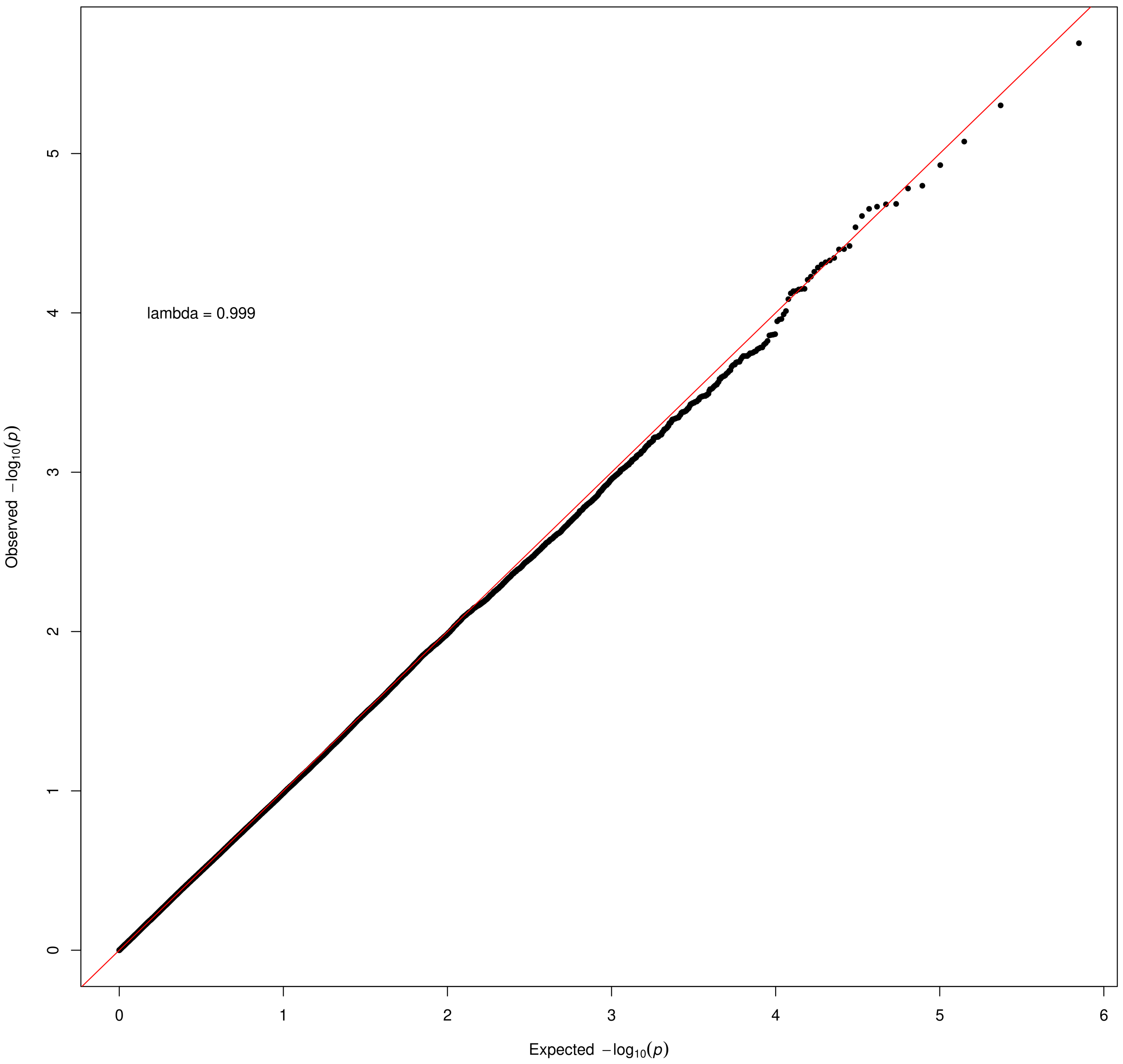

Supplementary Figure 12: QQ plot for lycopene, primary sample

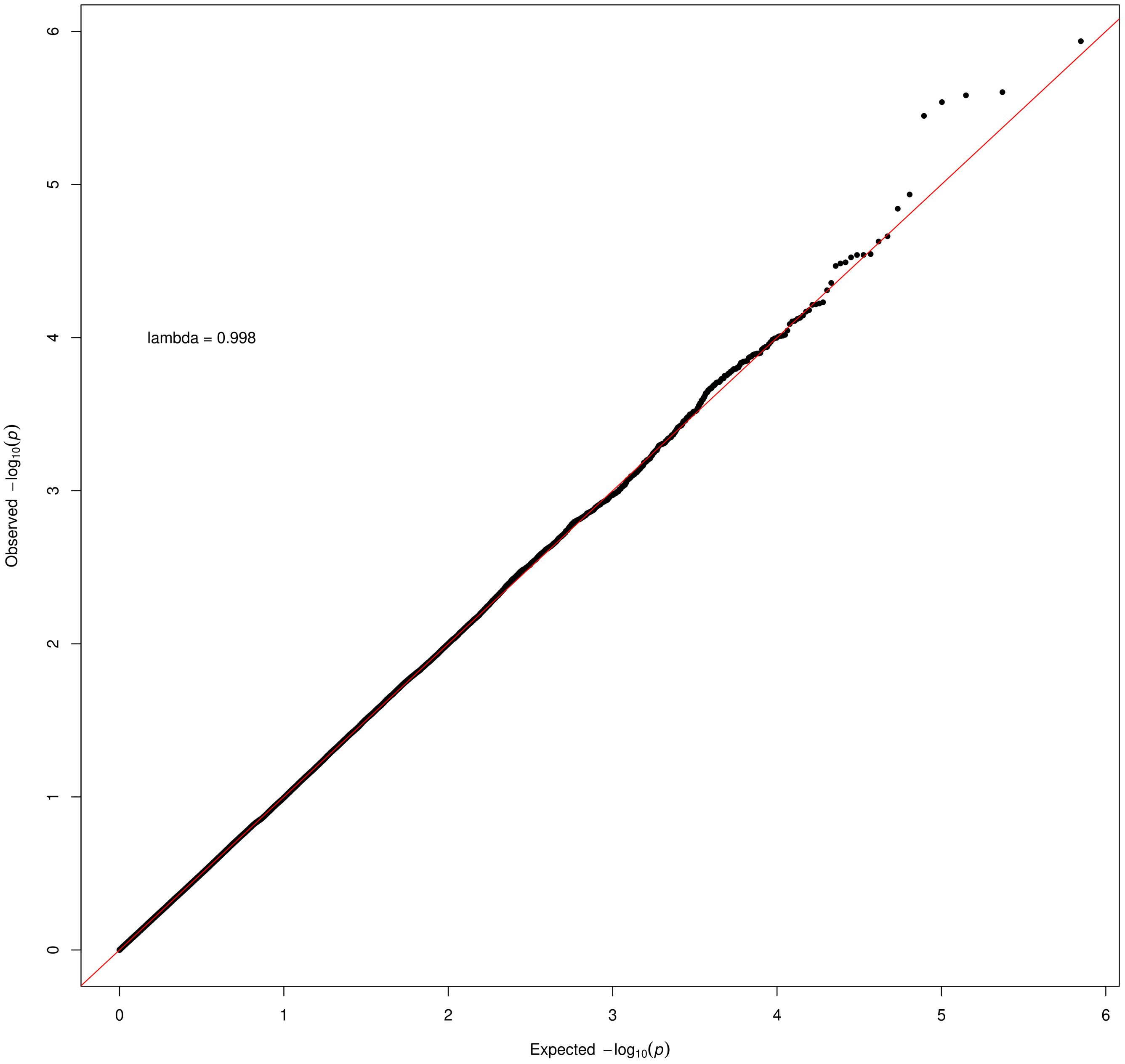

Supplementary Figure 13: QQ plot for cryptoxanthin, primary sample

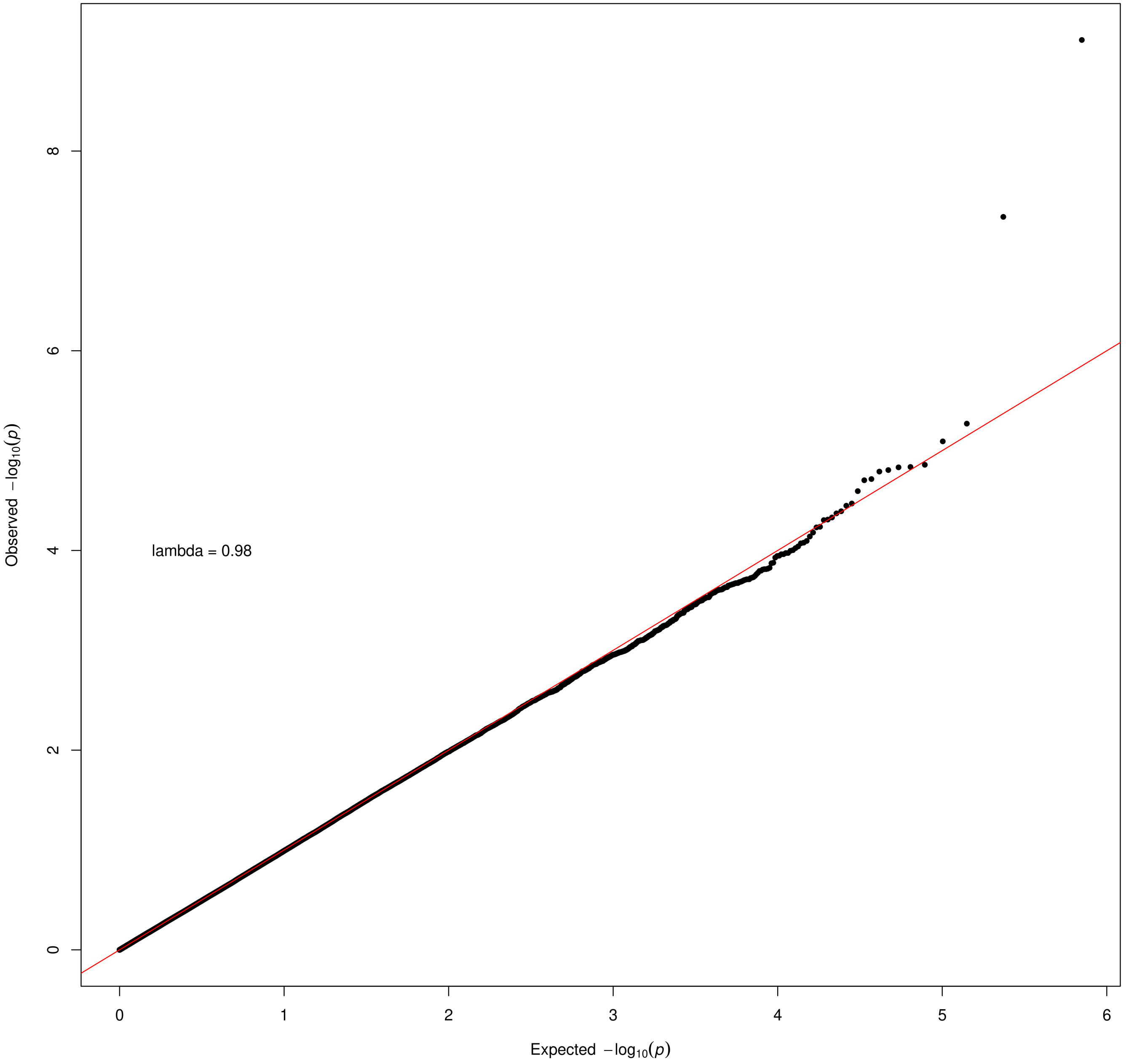

Supplementary Figure 14: QQ plot for Lutein, primary sample

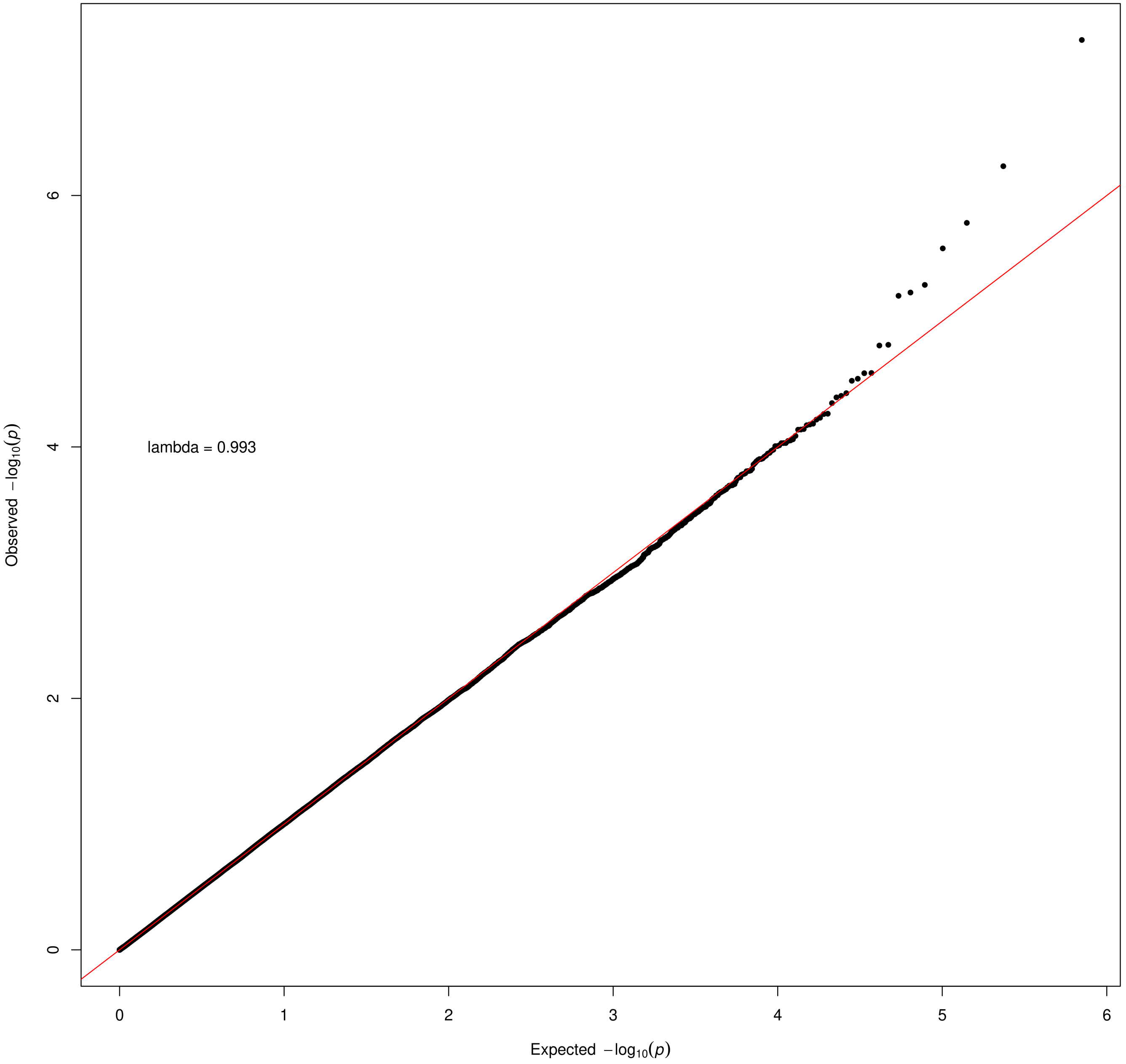

Supplementary Figure 15: QQ plot for Zeaxanthin, primary sample
